# EPS mid-career prize: An integrated framework for the learning, recognition and interpretation of words

**DOI:** 10.1177/17470218241284289

**Published:** 2024-10-12

**Authors:** M. Gareth Gaskell

**Affiliations:** Department of Psychology, University of York, York, UK

**Keywords:** Word recognition, complementary systems, lexical processing, language comprehension, consolidation, sleep

## Abstract

In this article, I review the evidence on the involvement of sleep and consolidation in word learning and processing during language comprehension, focusing on implications for theory. The theoretical basis for the review is a complementary systems account of word learning involving flexible (hippocampal) and stable (cortical) pathways to lexical knowledge. I argue that the accumulated data are consistent with a role for both pathways in both learning and recognition of lexical items, with sleep and consolidation supporting the transfer of recent experience between the pathways. The level of involvement of each pathway is dependent on key factors, such as consistency with prior knowledge in the case of learning, and reliance on context and/or automaticity in the case of recognition. As a consequence, the notion of a mental lexicon cannot really be restricted to just the listener’s stable knowledge about words: flexible knowledge and recent experiences are also important. Furthermore, I argue that the flexible pathway plays a critical role even in the absence of new lexical items. The available evidence suggests that this pathway encodes (and potentially consolidates) recent linguistic experiences, providing potential benefits to interpretation of subsequent language and the long-term retention of knowledge. In conclusion, I propose that a dual-pathway account incorporating both flexibility and stability is necessary to explain the learning, recognition, and interpretation of words.

## Introduction

The ability to acquire words and use them effectively is a crucial life skill. For example, good vocabulary at age 5 is associated with a reduced risk of mental health disorders into the teens and beyond (Beitchman et al., 2001; Schoon et al., 2010). Similarly, poor vocabulary in adulthood is associated with adverse life outcomes (Armstrong et al., 2017). It is also clear that word learning—even within a first language—is a lifelong undertaking, with on average one new word learned every other day between 20 and 60 (Brysbaert et al., 2016). The starting point for this article is the question of how word learning occurs. In other words, how do we acquire a mental representation of a new word that can sit alongside other word representations in the mental lexicon of the language user? There are a range of ways in which we can determine whether a new word has become part of the mental lexicon. Some obvious tests might be whether language users can define a that word when they hear or read it (testing receptive vocabulary), or whether they can name it from a picture (expressive vocabulary). Leach and Samuel (2007) described these factual tests of word learning as lexical configuration. However, to be a fully fledged lexical entry, the mental representation of a novel word would need to do more than this: it would need to interact with other lexical items and components of the language system during a conversation or other linguistic experience. For example, spoken word recognition involves a swift process of lexical competition (Marslen-Wilson & Tyler, 1980; McClelland & Elman, 1986), and so a potentially more demanding test of word learning would be whether a new word representation can take part in this competition process alongside more established lexical entries. Exhibiting this kind of property would be described as lexical engagement by Leach and Samuel, or as lexical integration in our own work (Gaskell & Dumay, 2003).

Does the distinction between configuration and engagement matter? A reasonable null hypothesis is that when people encounter new words, a new lexical representation is swiftly formed that would support both configuration and engagement equally. Such a position would be compatible with a wide range of theoretical positions, particularly if lexical representations are seen as sparse or single units in a localist connectionist network (Grainger & Jacobs, 2013; Page, 2000). However, beginning with our work on lexical competition, a body of evidence has emerged that suggests a more complex pattern of learning, with evidence of configuration appearing soon after exposure to a novel word, and (at least sometimes) engagement following after a delay. Gaskell and Dumay (2003) examined the acquisition of multisyllabic spoken words by teaching people pseudowords such as “cathedruke” that were selected to be close competitors of established words in the lexicons of participants (e.g., cathedral). Soon after repeated exposure to the novel words, participants were near-perfect in terms of their ability to recognise the novel items, given a two-alternative forced choice test consisting of the pseudowords and similar sounding foils (e.g., “cathedruke” vs “cathedruce”). However, despite this evidence of swift lexical configuration, the new words did not show any sign of influencing the recognition of existing words via lexical competition (tested using lexical decision or pause detection, Mattys & Clark, 2002). This lexical engagement effect (which manifested as slower responses to the existing words) was seen only after a delay, which in Gaskell and Dumay (2003) was a week later.

A similar delayed effect of lexical competition was seen for novel written words (Bowers et al., 2005; Clay et al., 2007), whereas subsequent auditory studies attempted to address the crucial factors that might lead to the emergence of lexical competition at a delay. Specifically, Dumay and Gaskell (2007) found that a 12-hr delay following exposure was sufficient to induce lexical competition effects for novel words, but only if that 12-hr period was overnight (and therefore included sleep). A similar period spent awake during the day was not effective in promoting lexical engagement, with these participants only showing competition effects at 24 hr, after sleep had occurred (see Wang et al., 2017, for parallel evidence in the case of written words). Furthermore, Davis et al. (2009) addressed the neural response to unfamiliar novel words, plus novel words learned immediately before and 24 hr before scanning using functional magnetic resonance imaging (fMRI). Consistent with the behavioural evidence, we found both swift and more delayed effects of word learning. The left hippocampus showed strong sensitivity to the initial presentation of unfamiliar novel words, and the strength of this response predicted memory for these items after scanning. Importantly, key cortical areas sensitive to lexical status (e.g., superior temporal gyrus) showed a nonword-like response to very recently learned words, but a more word-like response to the words learned 24 hr previously (see also Breitenstein et al., 2005; Gore et al., 2022; Tao et al., 2024).

Together, the early neurocognitive and behavioural evidence on word learning in adults suggested a theoretical account in which word learning occurs in two stages: first, an initial hippocampal encoding of newly encountered words that would support lexical configuration, and second, a more extended and slower consolidation process that would provide for lexical engagement. Although not the focus of the current article, there is a largely consistent picture emerging from related studies of word learning in development. Specifically, lexical competition effects tend to emerge or strengthen after a delay containing sleep for 8- to 10-year-olds (Brown et al., 2012; Henderson et al., 2012, 2013; Henderson & James, 2018). Naps consolidate word learning in 3-year-olds (Williams & Horst, 2014), and even for infants there is evidence of beneficial effects of sleep over wake (see Belia et al., 2023, for a review). Furthermore, studies of typical and atypical language development have suggested important individual and group differences in terms of both encoding and sleep-associated consolidation for novel words (Fletcher et al., 2019; Knowland et al., 2019; Smith et al., 2018).

In the next section, I will flesh out the above model, but then the bulk of the paper will be devoted to addressing how well this model fares in the face of a wider body of more recent evidence, mostly from adult participants. I should note that this article is quite wide-ranging in scope, and as a consequence cannot be a fully comprehensive review (cf. Duff et al., 2019; Palma & Titone, 2021; Schimke et al., 2021 for more focused reviews). I will argue that both learning and recognition of words are best understood in terms of the joint operation of both pathways in the complementary systems account, whereby the level of influence of each pathway depends on the nature of the material as well as the functional properties of the pathway. I will also argue that the range of phenomena that can be accounted for by this model is broader than first thought. In particular, the influence of recent linguistic material on upcoming comprehension can also be explained, regardless of whether the recent material contains novel lexical items.

## A complementary systems account of word learning

**Figure 1.**
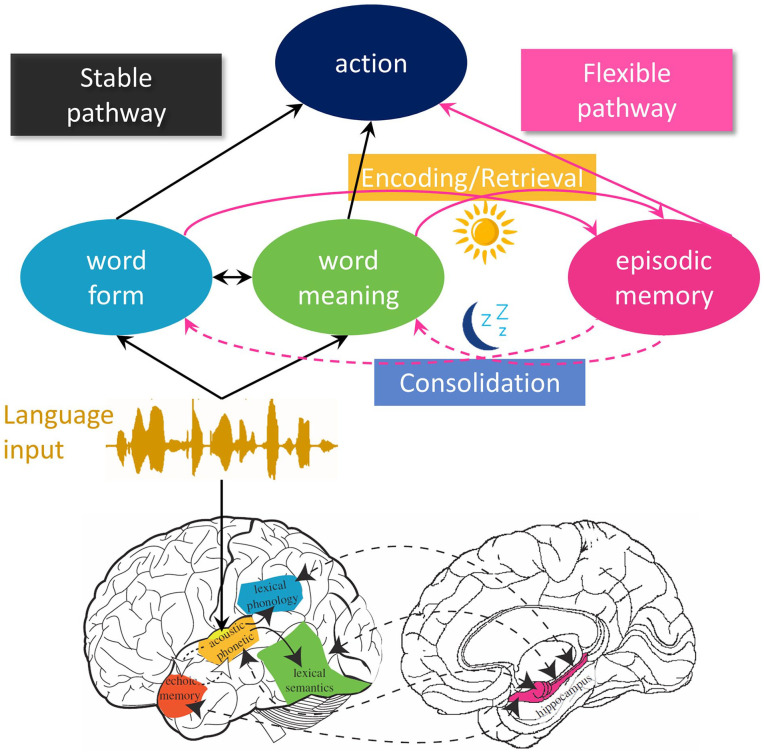
Architecture of the complementary systems account of word learning. In the upper section, the cognitive side of the model is sketched out. The black arrows mark the stable pathway making use of long-term lexical knowledge. The pink arrows mark the flexible pathway that exploits recent experiences in lexical processing. New comprehension events can trigger encoding in episodic memory. In turn, retrieval of relevant linguistic experiences from episodic memory can support interpretation. Lexical processing operates on the combined output of both pathways. During offline periods such as sleep, consolidation updates the stable pathway based on the contents of episodic memory (dashed arrows). In the lower section, the neural networks supporting the two routes are marked, with colours representing approximate correspondence with the cognitive elements. Lower section adapted from Davis and Gaskell (2009).

Davis and Gaskell (2009) laid out a descriptive theoretical account of word learning based on the evidence presented above (see Figure 1). Our starting point was an existing neural network model of human spoken word recognition (Distributed Cohort Model) that represents the recognition process as a mapping from a continuous stream of speech to distributed representation of the forms and meanings of the words embedded within that stream (Gaskell & Marslen-Wilson, 1997). When trained on a large corpus of language, this model learns words by gradually adjusting the connection weights between speech input and lexical output layers. However, once trained, the network cannot swiftly incorporate a new item that was not already in the original training set (unlike some of the localist models described above). If a trained lexical network was subsequently taught just the new word, the rapid readjustment of weights necessary to incorporate the new mapping would have the knock-on effect of overwriting existing mappings (i.e., other words) that rely on the same connection weights. This is the notion of *catastrophic interference* understood to be common to connectionist networks that make use of distributed representation (French, 1999; Mannering & Jones, 2021; McCloskey & Cohen, 1989). The fact that at least some configural aspects of word learning can be swift suggests that it cannot be solely based on such an architecture.

The problem of catastrophic interference applies to many models of memory and learning, and one highly influential solution is the complementary learning systems (CLS) framework (Kumaran et al., 2016; McClelland et al., 1995, 2020; Singh et al., 2022). Alongside a distributed, slow-learning, and relatively stable network such as the one described above, CLS theory posits that a second more flexible memory network can step in where required. This hippocampal network relies on sparser representations, and hence does not suffer from catastrophic interference. This makes it ideal for swift or even one-shot learning of new experiences that are dissimilar to stored representations in the distributed neocortical network. The other key element of CLS theory is that it allows for recent hippocampal memories to be integrated with the long-term neocortical memories through systems consolidation during offline states such as sleep. In these states, the hippocampal memory can be used to “teach” the cortical network the new memories in a slower and more interleaved manner, allowing for learning of the new material over time without loss of existing knowledge. More recent instantiations have gone into greater detail on how this teaching might take place, and how sleep might be involved in the transfer and integration process (Singh et al., 2022).

The attractions of such a solution applied to word learning are clear. The hippocampal network can be used to encode new words swiftly and easily, allowing for some configural properties to be acquired without any loss of information in the cortical network. Subsequently, sleep and other offline states can provide a means to integrate new words into the cortical network, providing aspects of lexical engagement such as the ability to influence lexical competition for established words, but only after a delay. The neuroimaging evidence from Davis et al. (2009) is also broadly compatible with this account, in that the hippocampus showed sensitivity to the very earliest stage of learning, whereas cortical areas that are sensitive to lexical status (word vs nonword) showed more word-like responses to the novel items after a consolidation period. At the time our review was written, evidence from polysomnographic recording of sleep was not available to address the question of whether sleep had an active role in consolidation of new lexical items, but a subsequent study (Tamminen et al., 2010) provided evidence that sleep spindles and slow-wave activity are associated with overnight behavioural changes in the lexical integration and accessibility of novel words. This fits well with a broader active systems consolidation theory of declarative memory during sleep (Born & Wilhelm, 2012; Klinzing et al., 2019; Rasch & Born, 2013).

As described, our complementary systems account of word learning is easy to interpret as being simple and dichotomous: a flexible episodic network encodes newly learned words, whereas established words are held separately and neocortically in the lexicon “proper.” Likewise, it might be tempting to infer from sleep studies such as Dumay and Gaskell (2007) that the consolidation process can take place over the course of a single night, leading to full lexical integration the following morning. But in both cases, this would be a misinterpretation, and we explicitly argued against such a view. First, relating to the time course of consolidation our point was that if sleep is involved in some way in the consolidation of lexical knowledge, then the first night of sleep would be the one to show the biggest change in neural representation (and thus behaviour), with gradually diminishing gains over subsequent nights. Second, relating to the separation or otherwise of the two systems, we argued that in fact the complementary systems account could be seen as incorporating two pathways between the same starting point (an acoustic-phonetic representation of speech input) and end-point (a distributed neural representation of phonological and semantic knowledge about the words in the speech input). Compared with the hippocampal pathway, the cortical route was seen as more direct (i.e., did not require hippocampal mediation) or otherwise prioritised in some way (e.g., faster, more automatic, or less effortful). This prioritisation of the stable cortical network was seen as an important aspect of the model, in that it would preserve the “optimal” balance of lexical competition between established words in the face of recent and unconsolidated experience with similar sounding words. But at the same time, the fact that both sides of the network eventually provide access to stored knowledge about words weakens the idea that true lexical knowledge relies on consolidated cortical representations of words. In effect, the distinction between configuration and engagement—while still highly relevant—becomes blurred when the inner workings of our model are interrogated. In the following sections, I will assess the properties of the two pathways further in the light of more recent evidence. The evidence comes from a wide range of methods, including both brain and behaviour, but with a bias towards behavioural evidence. To integrate this diverse evidence in a neurocognitive model, for shorthand I will refer to the cortical route as the *stable* pathway, accentuating its cognitive properties, and the hippocampal route as the *flexible* pathway.

## How much does the stable pathway contribute to encoding?

The characterisation of word learning given in Davis and Gaskell (2009) was clear in terms of the roles for the two routes in word processing. The flexible hippocampal pathway provided an opportunity to encode new words quickly without cost to the cortical network, whereas the stable cortical pathway provided a robust means of retaining and processing well-established words. As a neurocognitive model, there was also a more cognitive distinction between the two routes in that the flexible pathway was seen as more episodic in nature, whereas the cortical route was in some ways more abstract or semantic. While this characterisation remains relevant, more recent work has challenged a hard division between the roles of the two routes. In this section, I discuss potential ways in which some word learning might occur directly in the stable pathway, circumventing the episodic encoding and later consolidation of the flexible pathway. I will begin by reviewing evidence from patient studies, before turning to studies with both patients and healthy participants to address cases where the style of learning (e.g., fast mapping, implicit learning) and/or the nature of the learnt material (e.g., varying in consistency with prior knowledge) may influence the nature of encoding.

Given the key role of the hippocampus in our account, a crucial strand of evidence relates to cases where the hippocampus is compromised in amnesia (see Duff et al., 2019, for an excellent review). Briefly, it is clear from multiple studies (Elward & Vargha-Khadem, 2018; Gabrieli et al., 1988) that new learning of words and meanings is severely disrupted in amnesia, even for material that might be encountered repeatedly over a long period (Bayley et al., 2008). That said, there is also evidence that some new learning is possible, with the strongest evidence based on cases of developmental amnesia (Vargha-Khadem et al., 1997) where individuals with early-acquired hippocampal pathology showed competent spoken and written language abilities. It seems likely then that the hippocampus is important for typical word learning (at least beyond the first 18 months of life, Gómez & Edgin, 2016), but that other mechanisms can also operate in the absence of a functioning hippocampus.

What might be the alternative means of acquiring new knowledge when the hippocampus is damaged? Recall that the CLS theory was developed to solve the problem of catastrophic forgetting. However, catastrophic forgetting is not inevitable, depending crucially on the extent to which new mappings are consistent with existing knowledge (also known as schema consistency; Tse et al., 2007). If new mappings are inconsistent with prior knowledge, then indeed catastrophic interference will occur. However, McClelland (2013) demonstrated that new mappings that overlap substantially with prior knowledge can be learned relatively quickly in a single cortical network without causing substantial interference to the retrieval of the prior knowledge. Essentially, the new learning can occur by “tweaking” existing representations, meaning that there are no wholesale changes in connection weights that would impact on prior memories. This point is potentially relevant to one clear example of new label learning in adult hippocampal amnesia (Duff et al., 2006). In their study, patients had to describe tangrams (abstract shapes) across a series of trials in a referential communication game with a familiar partner. Over the course of several trials, descriptions became briefer and more like new labels for the ambiguous shapes. Furthermore, these labels were consistently retained and used over long periods (6 months to 2 years). Importantly, though, the participants exploited their prior knowledge in settling on labels, with the example given of a figure that could be interpreted as a man lying against a tree being referred to as “siesta man”). This fits with the notion that some hippocampally independent learning of words can take place for mappings that overlap with existing lexical knowledge.

Potentially, a similar argument can be made for the phenomenon of “fast mapping” in amnesia (Sharon et al., 2011). Sharon et al. tested hippocampal amnesia patients using two types of learning trial. In explicit-encoding trials, participants saw a single unfamiliar picture (of an animal, fruit, vegetable, or flower) and were explicitly told to link it to a novel word form (e.g., “remember the mangosteen”). This type of learning was compared with fast-mapping trials inspired by developmental studies of early word learning (Carey & Bartlett, 1978). In these, there was again a novel word form and a picture of an unfamiliar item, but this was presented in the context of a familiar item from the same semantic category (e.g., a zebra), and participants had to answer a question about the novel item (e.g., “Is the numbat’s tail pointed up?”). In this kind of trial, there is no direct instruction to encode the novel form meaning mapping, but the participant can infer the intended referent from the comparison with the known item (e.g., the numbat can’t be the zebra, so it must be the other animal). This kind of learning is highly effective in studies of infant learning in terms of initial association between form and meaning, although good retention in the long term may rely on repeated exposure (Horst & Samuelson, 2008). For the patients, as expected, explicit encoding led to poor retention 10 min and a week later, as compared with matched controls. However, in the fast-mapping condition, the patients’ performance was substantially better at both time points: their performance remained above chance a week after the learning trials and was not significantly different from the control performance. This study provides further preliminary evidence that words can sometimes be learned via cortical mechanisms.

It is worth considering whether fast mapping can make better use of prior knowledge than explicit encoding, in which case it could potentially be explained within CLS theory as described above. Certainly, the relationship between form and meaning in these trials is essentially arbitrary, suggesting not. But, nonetheless, the availability of a known, semantically similar, lexical item in fast-mapping trials, and the implicit instruction to relate the new items to those existing semantic neighbours might provide more in the way of existing schematic knowledge (i.e., similarity in representation) to scaffold the new learning in accordance with a CLS account (cf. the “siesta man” example above). Such an explanation might predict that patients would learn the new words relatively weakly, which does not fit with the initial finding from Sharon et al. (2011). Importantly, though, subsequent studies have not found robust retention for fast mapping in hippocampal amnesia, instead finding either retention in specific but limited circumstances (Merhav et al., 2014) or little or no evidence for any retention at all (Cooper et al., 2019; Warren & Duff, 2014; Warren et al., 2016). Interestingly, adults with developmental amnesia also showed no evidence of fast mapping benefitting retention of word meanings as compared with explicit encoding (Elward et al., 2019). Therefore, the broader body of evidence is reasonably consistent with the notion that fast mapping is not a mechanism that can bypass hippocampal word learning if robust and generalisable memory traces are required. Furthermore, to the extent that some retention is possible for fast mapping in amnesia, the results are potentially consistent with a CLS interpretation relating to the prior knowledge benefits associated with the learning context.

The above studies can all be described as addressing lexical configuration (i.e., can the learner identify the meaning of the new word). However, if fast mapping really does circumvent the need for hippocampal mediation, it should also allow for lexical engagement effects without sleep. This was an intriguing question addressed by Coutanche and Thompson-Schill (2015), who assessed explicit encoding and fast mapping in terms of their ability to induce new visual lexical competition effects in healthy adults using the method developed by Bowers et al. (2005). Their critical finding was that for fast-mapped but not explicitly encoded words there was a lexical competition effect evident soon after learning, fitting with the idea that these items had become lexically embedded without the need for hippocampal mediation and consolidation. However, just as with the evidence on fast mapping in amnesia, the replicability of this finding is questionable (Gurunandan et al., 2023), suggesting that if this effect is real, it may be fragile or quite restricted to the detailed experimental conditions of the original study. Indeed, currently unpublished data from our own lab also failed to find lexical competition effects from fast mapping, whether tested visually or auditorily (see Gaskell & Lindsay, 2019).

A parallel debate has addressed the gradual learning of repeated sequences of items such as syllables embedded within a longer nonrepeating stream. This form of learning (known as the Hebb effect; Hebb, 1961) is generally seen as implicit as it can occur without awareness, and it can be observed in patients with hippocampal amnesia. For example, Gagnon et al. (2004) used sequences of digits, words, and pseudowords in a serial recall task. In the majority of trials, the order of these items was random, but on every third trial, the same fixed-order list was presented. Although the participants were not aware of this manipulation, both the amnesic patient and matched controls performed better on the fixed-order list than the random lists, suggesting some form of learning had taken place. Might this kind of learning be a good model for cortical word learning, particularly in cases where words (and particularly their meanings) are derived from encountering them in sentence context? Szmalec et al. (2012) tested this by looking to see whether trisyllabic novel words learned in a Hebb paradigm (e.g., the syllable sequence “sa-fa-ra”) might induce lexical competition for existing spoken words (e.g., safari). Their results were intriguing in that competition effects were not found soon after learning, but unlike Dumay and Gaskell, they were found after a 12-hr delay regardless of whether or not that delay contained sleep (i.e., the effects were equivalent after AM-PM delay and a PM-AM delay). This lack of a sleep–wake difference might suggest that the learning seen in the Hebb effect is not subject to sleep-associated consolidation effects despite some consolidation during wake *or* sleep still being needed (although cf. Fernandes et al., 2009). Once again, though, the story is not so simple. In four experiments, Sobczak and Gaskell (2019) attempted to conceptually replicate Szmalec et al., while comparing Hebb learning with more explicit learning of novel words (exposure to the novel sequences in a phoneme monitoring test). While the more explicit learning led to lexical competition effects after a suitable level of exposure and an interval including sleep, the Hebb condition showed no significant lexical competition effects at any point before or after sleep. Again, this failure to replicate does not imply that lexical engagement is not possible using a learning style similar to the Hebb paradigm, but it does suggest that learning in this manner might be fragile or dependent on a set of specific conditions.

The above studies suggest that circumstances in which the hippocampus is not implicated in word learning tend to lead to weak and/or hard to replicate learning effects, implying that this means of acquiring new vocabulary is possible but far from optimal. Exceptions exist (e.g., Duff et al., 2006), providing some hints that prior knowledge or consistency with existing schemas can support cortical learning. However, this interpretation is post hoc and would benefit from more direct evidence relating to the benefits of schema consistency. In the broader memory literature, this evidence is available, with both animal and human studies suggesting that schema-consistent information is better learned via cortical routes (e.g., Kan et al., 2009) and relies less on subsequent consolidation to become hippocampally independent (Tse et al., 2007).

Building on standard systems consolidation theories, van Kesteren et al. (2012) argued for a key role of the medial prefrontal cortex (mPFC) in explaining schema consistency effects. In their SLIMM (Schema-Linked Interactions between Medial prefrontal and Medial temporal regions) model, the mPFC acts as a triage and gating hub. When new experiences are perceived, the mPFC detects the congruency of the new material with existing cortical knowledge through a resonance process. In the case of weak resonance (for a schema-inconsistent experience), the medial temporal lobe (MTL) steps in and forms a new episodic memory for the experience. However, if the new experience resonates strongly with existing cortical knowledge (suggesting that the new experience is reasonably compatible with existing cortical memories), reciprocal links between mPFC and MTL inhibit episodic encoding, and instead cortical learning is potentiated. This theory also suggests that there might be a middle ground in consistency with prior knowledge such that neither MTL nor cortical networks are strongly activated, which can explain the u-shaped nature of prior knowledge effects on memory (Quent et al., 2022).

If the engagement of the hippocampus for new memories is dependent on prior knowledge consistency, then by extension one would expect sleep effects on consolidation to be similarly affected. That is, for new memories that are strongly schema consistent, the bulk of the learning will be cortical and there will be little or no episodic memory to be consolidated during sleep. In contrast, for schema-inconsistent new memories, the bulk of the encoding will be hippocampal and so sleep should be needed to help consolidate memories post-encoding. Although it is hard to specify exactly where new memories sit on the continuum of schema consistency, one clear advantage of language in this respect is the wealth of paradigms that can be exploited to address variables such as systematicity and consistency in natural and artificial languages.

To this end, Mirković and Gaskell (2016; see also Mirković et al., 2019) used an artificial language that varied in the extent to which different elements exhibited systematicity/arbitrariness. Participants had to learn form-meaning mappings for two-word sequences paired with a picture of the referent (e.g., *tib scoiffesh* might refer to a picture of a ballerina). These sequences contained stem forms (e.g., *scoiff*) that were arbitrarily related to the meaning given to that word (i.e., ballerina). At the same time, the first word in the sequence and the suffix of the second word both signalled the grammatical gender of the sequence, which in this case fully overlapped with the natural gender of the depicted referent (e.g., *tib* + *-esh* sequences were designated as feminine gender and were associated with images of women). Participants were all native English speakers and so did not use grammatical gender in their native language. During the course of learning to associate word sequences with pictures, there were elements of learning that could not rely on prior knowledge of other associations (e.g., learning that *scoiff-* refers to a ballerina is not helped by knowing that *heef-* refers to a cowboy). However, the grammatical elements of the mapping were shared across many different learning trials and so exhibited consistency within that language. Assuming that each trial leads to a very small amount of cortical learning (as the CLS predicts), over time, structure would be built up in the cortex that would be more helpful to the systematic aspects of the language learning than the arbitrary aspects. If SLIMM is correct, this would imply that the arbitrary components of the language would have the strongest hippocampal representation after learning, and so the most likely components show a benefit of sleep over wake. This is exactly what we found. When tested after a nap or an equivalent period awake, tests that relied on specific arbitrary knowledge about the stem forms showed better performance in the sleep group than the wake group, whereas tests of the more systematic knowledge showed no such benefit. The chain of interpretation for this behavioural experiment on healthy adults is undoubtedly longer than for the studies of patients, but the result is nonetheless compatible with the notion that the cortical network can aid word learning, but its effectiveness depends on the properties of the mapping, with systematic aspects more suitable to cortical learning than arbitrary aspects (see also James et al., 2019, 2020, 2021 for developmental evidence along similar lines).

Based on evidence from an array of diverse methods in healthy adults and patients, there is a reasonably consistent neurocognitive interpretation of performance for word learning. The hippocampus and MTL structures support the bulk of word learning, meaning that damage to that route severely impairs the ability to acquire new words. That said, various sources of evidence suggest that cortical learning is possible, albeit weakly or in specific circumstances. The simplest interpretation of these exception cases is that overlap with prior knowledge is the key to cortical learning, with a potential role for the mPFC in assessing the degree of overlap and thus the response of the hippocampus (van Kesteren et al., 2012). Where the hippocampus needs to be strongly engaged, consolidation effects are likely to be seen, associated with sleep, whereas pure cortical learning may need little or no consolidation.

## How much can the flexible pathway contribute to lexical processing?

As discussed, the word learning theory laid out by Davis and Gaskell (2009) was careful not to make the simple assumption that hippocampal learning of words would lead only to lexical configuration effects and that cortical integration via consolidation was needed for lexical engagement. Rather, we saw the distinction in terms of a prioritisation: both routes provide access to lexical knowledge, but with the stable cortical pathway being more direct or obligatory or faster, such that hippocampally mediated knowledge was unable to induce lexical inhibition when well-established words are recognised (e.g., the recognition of *cathedral* when “cathedruke” is heard). That said, there remained a tendency in our work (e.g., Dumay & Gaskell, 2012) and others (e.g., Qiao et al., 2009) to think of episodic memories of words as somewhat peripheral and/or not proper lexical items. Increasingly, this “purist” vision of the lexicon has become untenable, with a more holistic view of the lexical representation incorporating both episodic and abstract components becoming dominant (cf. Pufahl & Samuel, 2014).

The extent to which flexible and stable pathways contribute to different tests of lexical status remains an open question with this more holistic approach to lexical representation. Our work in this area has been behavioural and has operated on the assumption sketched out in the last section that most initial word learning will be hippocampally mediated, and that substantial cortical representation will typically only emerge after a consolidation period including one or more periods of sleep. Based on these assumptions, we assessed an array of tests of lexicalisation to determine whether novel items performed as if they were a word before (largely via the flexible route) or only after sleep (at least to some extent via the stable route). I will discuss a sample of this evidence, with a focus on whether consolidation leads to more automatic access to lexical knowledge, as well as addressing studies that have used the visual world paradigm to assess word learning.

Our suggestion that the stable pathway to lexical knowledge might have some form of priority or advantage was highly speculative, based on the observation that newly learned words tend not to engage in lexical competition initially (when held in the flexible pathway) but tend to show competition effects after sleep (when partially consolidated in the stable pathway). To try to make this notion of priority more concrete, two studies assessed aspects of automaticity in language processing, with the idea being that automatic recognition of novel words might be more reliant on the stable route and so preferentially observed post-consolidation. Of course, automaticity itself is a complex concept. It is multifaceted (Moors & De Houwer, 2006), and unlikely to be underpinned by any one memory system (Ashby & Crossley, 2012). Nonetheless, it is feasible that cortical consolidation of words might at least enhance the level of automaticity associated with recently learned words. Tham et al. (2015) tackled this question by assessing two markers of automaticity in word processing: the size-congruity effect and the semantic distance effect (Rubinsten & Henik, 2002). Native English–speaking participants learned written Malay and Mandarin words that referred to familiar animals of different physical sizes (e.g., *bee*, *cow*, *dog*). Twelve hours later after a period awake or including sleep, they were presented with pairs of newly learned words and asked to make judgements about either the physical size of the referent or the font size of the presented words. In the context of this study, a size-congruity effect would be a response time benefit for stimuli that match rather than mismatch in terms of relative referent and font size, and a semantic distance effect would be a response time benefit for judgements of referent size that are substantially different in size over finer judgements (e.g., *cow-bee* vs *cow-dog*). The results were complex, but fitted with the idea that some aspects of automaticity are observed prior to consolidation but that sleep-associated consolidation enhances the strength of automaticity effects and extends the range of indicators of automaticity. In particular, the size-congruity effect, which involves interference from an unattended dimension, was not observed in the wake condition, but did emerge (in some circumstances) after sleep. These enhancements were correlated with slow-wave sleep and sleep spindle activity, fitting with the broader literature on sleep effects on memory consolidation (Rasch & Born, 2013) as well as our own prior work on lexical competition for new words (Tamminen et al., 2010).

Along similar lines, Geukes et al. (2015) assessed the involvement of sleep in the classic measure of automaticity, the Stroop test. Native German–speaking participants learned 10 novel words that were associated with German colour terms. After a delay of 2 or 24 hr, they were required to make judgements about the font colour of the novel words as well as their German equivalents, with the font colour either matching or mismatching the meaning of the word. The key question was whether or not the knowledge of the meaning of novel colour term would interfere with the font colour judgement, as would occur for a well-established colour term. The results were intriguing, and in some ways similar to the Tham et al. (2015) study. If the novel words were presented at test intermixed with the familiar German words, then the typical Stroop effect of slower responses for mismatching trials was found regardless of whether the delay period included sleep. However, if the novel words were presented at test without the context of the familiar words, then Stroop interference was only observed after a delay including sleep (i.e., 24 hr). The combined influences of both a consolidation period and context are particularly pertinent to the issue at hand. Taking first the influence of a 24-hr consolidation period, it seems that this is enough to support Stroop interference from the novel word meaning regardless of the contextual circumstances. By our account, this is because consolidation has resulted in a cortical representation of the novel word, allowing access to its meaning quickly enough (or in a more obligatory way) to influence the judgement of the font colour. In other words, the (partially) consolidated words were behaving as you would expect lexically well-established words to behave. Prior to consolidation, this cortical representation was presumably not well enough established to generate Stroop interference, but of course there would still be a strong hippocampal representation. In the absence of contextual cues to meanings (i.e., the German colour words), this representation did not seem to be sufficient to activate the word meaning effectively, but if the encoding context was also present at test, then this seemed to have the effect of facilitating retrieval of the episodic memory such that Stroop effects can be seen. This fits with the broader understanding of the role of the hippocampus in binding item and context in episodic memory (e.g., Ranganath, 2010).

The visual world paradigm has been used extensively to track the time course of lexical competition in spoken word recognition (e.g., Allopenna et al., 1998; Apfelbaum et al., 2023). Participants are asked to listen to spoken words or brief phrases while viewing a small set of images on a screen that might relate in different ways to the spoken input. The record of fixations to the images as the speech unfolds provides a rich measure of the lexical hypotheses entertained at each point in time. For example, Allopenna et al. found that when listening to a word (e.g., *beaker*), both onset-matching competitor objects (e.g., *beetle*) and rhyme-matching objects (e.g., *speaker*) would be fixated more than control objects. This shows that lexical competition effect can be observed using the visual world paradigm. With respect to newly learned words, Kapnoula et al. (2015) taught people novel competitors to monosyllabic existing words (e.g., *jod* for *job*). They then used the visual world paradigm to assess whether this familiarisation might show competition effects during the recognition of the existing word. Strikingly, this competition effect was indeed seen (i.e., fewer looks to the existing word image) soon after learning and without a consolidation period, even though there was no picture associated with the novel word during learning. The time course of the novel item competition effect was very similar to the time course seen for an existing competitor (e.g., *jog*). As such, the evidence from the visual world paradigm (see also Kapnoula & McMurray, 2016; Weighall et al., 2017) provides evidence for lexical engagement prior to consolidation, in stark contrast to the majority of evidence from other tests addressing lexical competitor such as pause detection, lexical decision, or word-spotting (Dumay & Gaskell, 2012; Gaskell & Dumay, 2003).

One interpretation of this discrepancy might be that the visual world paradigm is simply more sensitive than the other tasks mentioned, and so is able to capture weak cortical learning, as in the previous section. However, another possibility is that episodic memories are more important in terms of the operation of the visual world paradigm than other paradigms. In the Kapnoula study, as is standard for the visual world paradigm, there was a preview period before each trial that allowed participants to look at the pictures ahead of encountering the spoken stimulus (lasting 500 ms plus the time taken to observe a blue circle and click on it with a mouse). This preview is important because it allows the listener to set up a mental model of the images, avoiding the need for a visual search during the spoken word perception. Indeed, following a preview period image can be removed from the screen and target-related fixations will still be observed (Altmann, 2004). It is possible that a consequence of the preview period is that a representation of the target word (e.g., *job*) is built up in episodic memory, including some representation of its spoken form. If so, then this would have a competitor item also in episodic memory (i.e., *jod*) and that competition for retrieval from episodic memory drives the delay in fixating the target word. This would set the visual world paradigm apart from tasks such as pause detection or lexical decision (e.g., Gaskell & Dumay, 2003) where no preview period is available, and the existing word competitor is unlikely to be in episodic memory. In short, my argument is that we are likely to see competition effects if both new competitor and existing word are represented in the same pathway. In the case of our original studies of word learning, this would be the case in the stable pathway after consolidation. In the case of the visual world paradigm, my contention is that both are represented in the flexible pathway, due to the training on the novel word and the presence of a preview period.

This interpretation of the visual world paradigm, like our interpretation of the contextual version of the Stroop paradigm described above, highlights the importance of the retrieval process from episodic memory in that it must be a competitive process based on the relevance of the memories to the current situation/context. This view sits well with current models of episodic encoding and retrieval that stress the competitive element of retrieval (Lu et al., 2022). My argument is that certain experimental situations (e.g., the contextual Stroop or the visual world paradigm) will lean heavily on the episodic memories in the flexible pathway, whereas others (e.g., the classic Stroop paradigm or lexical competition as tested using pause detection) cannot. As such, it can be argued that neither the visual world paradigm nor pure auditory tasks provide the “correct” view of lexical competition, more that both in combination provide a complete view of how listeners make use of their combined sources of information about lexical items in different circumstances.

While necessarily selective, the evidence from this section hopefully gives a flavour of the potential for both flexible and stable pathways to support the recognition and use of new words in a CLS account. Prior to a consolidation opportunity, episodic hippocampal representations of new words typically provide a solid foundation for lexical representation and contribute to an array of behaviours when lexical knowledge is tested. As consolidation progresses (over days, weeks, and perhaps months), the stable cortical representations of words get stronger, and the hippocampal representations likely decay. Although no single study provides a definitive understanding of the consequences of this shift, in combination, they suggest that lexical representations become less contextual, more readily accessible, more automatic, and better integrated with long-standing lexical knowledge. Nonetheless, it is clear that flexible route representations contribute much to our ability to evaluate and manipulate words in conversation.

## What is the scope of a complementary systems account?

Davis and Gaskell (2009) presented a model that was squarely focused on the issue of how we acquire new words and add them to the mental lexicon. While it necessarily dealt with questions of processing (e.g., engagement in lexical competition), this was to understand properly the learning process. However, as further evidence has accumulated, we can see that the framework laid out in that paper potentially does much more. While the details of the framework remain incomplete, we can see that both pathways in the model contribute to both learning and recognition of spoken words. The brunt of novel word learning is borne by the flexible hippocampal pathway, but the stable cortical pathway may also be involved depending on compatibility with prior knowledge. Similarly, the brunt of recognition of learnt words is borne by the cortical pathway, but the hippocampus may also be involved in a time-limited way. Evidence from a host of psycholinguistic tasks is best explained as a consequence of both routes working cooperatively. This is a quite different way of thinking about word processing, in that other models of human word recognition have exclusively focused on stable knowledge of lexical items (e.g., Coltheart et al., 2001; McClelland & Elman, 1986; Norris, 2006). In this article, I have argued for embracing plasticity in lexical processing, so that recently acquired words can take part in lexical processing alongside more established knowledge.

Given this shift in emphasis, it is reasonable to ask whether there is more that the flexible system can provide in terms of lexical processing beyond the learning of new words. If recent experiences are part and parcel of lexical processing, then do those experiences need to involve a novel word to be influential? Although word learning is reasonably common throughout adulthood (Brysbaert et al., 2016), the vast majority of sentences encountered by an adult will not contain novel or even recently learned words. Is the flexible route inactive in those circumstances? A new body of evidence would suggest not, providing support for a model in which the flexible route is potentially always active, encoding recent linguistic experiences to support the optimal interpretation of upcoming language.

## Incorporating recent experience in lexical processing

A key paradigm that has been used in understanding how recent experience might affect word recognition is word-meaning priming, developed by Rodd and colleagues (Betts et al., 2018; Rodd, 2020; Rodd et al., 2013). The focus of the paradigm is the recognition and interpretation of words with multiple unrelated meanings (i.e., homonyms) such as *pen*, which could mean a writing instrument or an animal enclosure. Proficient language users are able to resolve this ambiguity with relative ease, although there is also sensitivity to the relative frequency of the different meanings, with less common subordinate meanings (e.g., the animal enclosure meaning of *pen*) being a little more difficult to access (Duffy et al., 1988). This prioritisation of more common meanings makes sense from a processing efficiency point of view, but also implies that language users must acquire the frequency statistics of the various meanings of an ambiguous word to boost the more frequent interpretation.

Rodd et al. (2013) assessed whether a single experience with an ambiguous word in a sentence context that supported its subordinate interpretation might have observable consequences at a later timepoint. Participants listened to and made meaning judgements about sentences that contained ambiguous words, with the sentence always supporting the subordinate meaning (e.g., “The farmer moved the sheep into the *pen*”). They were given an unrelated filler task, before encountering the ambiguous words again, this time in isolation in a word-association test. The associates were then categorised in terms of whether or not they related to the subordinate meaning. The central question was whether, about 20 min after the sentence exposure, participants were more likely to retrieve an associate that was related to the subordinate meaning, in comparison with a control condition for which the sentence exposure was withheld. Rodd et al. found that this was the case, with about a 7% boost in the percentage of associate responses that were related to the subordinate meaning in the primed condition.

Subsequent studies replicated and extended this work, with an overall pattern of performance suggesting that word-meaning priming could survive an interval of a few hours, but with gradually declining strength (Rodd et al., 2016). Importantly, word-meaning priming is relatively abstract, in that priming across modality (i.e., auditory-visual or visual-auditory) is not significantly weaker than priming within modality (Gilbert et al., 2018). At least initially, this priming effect was interpreted with reference to the need to collect and retain lexical statistics about meaning frequency for ambiguous words as mentioned above. In other words, an exposure sentence was seen as a new incidence of the ambiguous word being resolved towards the less common meaning, leading to an adjustment of meaning strength with the subordinate meaning now slightly less dispreferred. In the context of the complementary systems account described in this article, this would be explained in terms of a modest adjustment of weights in the stable cortical network, subtly shifting the balance of meanings in terms of their accessibility. This *immediate adjustment account* (Gaskell et al., 2019) makes a lot of sense in that there is ample opportunity to build on prior knowledge of the meaning of the ambiguous word, meaning that cortical learning is feasible in a complementary systems model. That said, there are also certain questions that this kind of explanation would raise. First, it is harder to explain why the strength of word-meaning priming might decay in the minutes and hours following exposure if the effect of exposure is to make a lasting change to the weights in the lexical network. Furthermore, it is perhaps surprising that a single exposure to a subordinate meaning would lead to quite substantial shifts in the accessibility of that meaning, given that the overall balance of probabilities amassed over a lifetime of language will have changed only slightly.

In the light of the complementary systems account fleshed out in the current article, an alternative explanation is worth considering. Although it is certainly feasible that encountering an ambiguous word with a particular meaning could lead to selective strengthening of that meaning in the cortical lexical network, it is also possible that an episodic memory is formed via the flexible route. The ambiguous word in itself is not a novel item, but nonetheless the contextual associations of that word are much more likely to be novel or at least very rare. Therefore, it may be that when we encounter a sentence, an episodic representation of the sentence is formed, including a contextually bound representation of the ambiguous word. Furthermore, just as episodic representations of novel words can contribute to lexical processing at a later timepoint, these sentence representations could contribute to the interpretation of the same ambiguous word later on alongside the cortical knowledge, providing a biasing source of evidence towards the subordinate meaning. This *episodic context account* (Curtis et al., 2022) can be distinguished from the immediate alteration account in at least two ways. First, it can accommodate the drop-off of word-meaning priming over time as a consequence of hippocampal trace decay during wake (Hardt et al., 2013). Second, it would predict that sleep should preserve these episodic memories over wake, and potentially provide a means of integrating the new contextual knowledge about the words into the cortical lexical network.

Gaskell et al. (2019) tested the sleep consolidation prediction in two word-meaning priming experiments. The first experiment made use of exposure and test sessions as above, but extended the interval in between to 2 hr in the afternoon, or 12 hr either overnight or during the day. This first experiment found that word-meaning priming remained robust across sleep periods (a 2-hr nap or 12-hr overnight) but diminished across wake (2 hr awake in the afternoon or 12 hr across a day). This result fits with the notion that sleep preserves episodic memories of the exposure sentences, as predicted by the episodic context model. However, it could also be explained in terms of the potential interference effect of intervening language exposure, given that wake periods will typically contain more linguistic interaction than sleep periods. To address the latter explanation, a second experiment extended the period between exposure and test to 24 hr, with both sessions occurring either in the evening or in the morning. These two conditions should be equated on overall linguistic interference across a day and a night but differ in the timing of the consolidation opportunity (early vs. late). Given that 12 hr awake subsequent to exposure eliminated word-meaning priming in Experiment 1, there was no expectation of a priming effect after 24 hr when participants started the experiment in the morning, and none was found. The real question was whether word-meaning priming would survive 24 hr when exposure was in the evening. If the sleep period had supported consolidation of the episodic memories, then these should then be more robust to interference during the day following sleep (Gais et al., 2006). However, if sleep just provides a passive barrier to interference from subsequent language, then a day awake following sleep should eliminate word-meaning priming. The results were consistent with the consolidation account, again supporting an episodic context account of word-meaning priming.

The results of Gaskell et al. (2019) suggest that listeners in some circumstances will form new episodic memories of sentences that are susceptible to consolidation during sleep and can inform the interpretation of constituent words if they are encountered at a later point in time, alongside the cortical body of lexical knowledge. The study left unknown exactly what would trigger the episodic encoding of a sentence memory of this type. One possibility is that encoding occurs automatically during comprehension, as part of the process that supports language interpretation and retention across longer periods of time. Alternatively, it could be that aspects of the experimental setup encouraged encoding, such as requirement of a meaning judgement following exposure, or the knowledge that participants will be required to return for a second session. Nonetheless, it seemed unlikely that the presence of a homonym in each of the exposure sentences was a trigger to encoding, given the ubiquity of lexical ambiguity in language. Therefore, two subsequent studies set out to test the generality of word-meaning priming for different types of words.

Curtis et al. (2022) addressed the question of whether word-meaning priming is specific to homonyms or can be found more generally. We used words that are not classed as homonyms (e.g., *balloon*) and sentence contexts that highlighted specific aspects of that word’s meaning (e.g., “The entertainer filled the balloon from the gas cylinder and inhaled it to make her voice squeaky”). As in Rodd et al. (2013), a word-association test about 20 min later allowed assessment of whether the exposure sentence had influenced interpretation of the isolated word. In this experiment, there was also a speeded semantic decision test as a second measure of word-meaning priming, in which the target word was paired with a word that was related to the exposure sentence (e.g., *balloon-helium*). Across three experiments, the results showed that word-meaning priming does indeed occur for non-homonyms and is consistent with the proposal that language comprehension routinely involves the encoding of contextually bound representations of words. Interestingly, the same kind of priming effects were also found for a condition in which the exposure sentence did not contain the target word, but did contain a closely related word (e.g., replacing *balloon* with *vessel*). This semantic priming effect was unexpected (and conflicts with evidence from Rodd et al., 2013), but fits with the understanding that precise memories of the words in a sentence are forgotten quickly (e.g., Fisher & Radvansky, 2018), allowing some generalisation of word-meaning priming to strongly related words. Alongside the evidence that modality of exposure is not important in classical word-meaning priming (Gilbert et al., 2018), this suggests that the contextual memories formed during comprehension are somewhat abstract. This represents a step back from a “pure” episodic view of sentential encoding in which memories are highly detailed in terms of form (cf. Goldinger, 1998), but is quite compatible with a body of current thinking on the relationship between episodic and semantic memory (cf. De Brigard et al., 2022).

A further study (Mak et al., 2023a) took this more generalised form of word-meaning priming and tested whether it held more strongly across sleep than wake, using a design similar to Gaskell et al. (2019; Experiment 1). A protective effect of sleep was indeed found for the word-association test, although the results for the speeded decision were less clear. A second experiment assessed priming using words that were ambiguous as to their word class (e.g., *loan* can be used as a noun or a verb, although in this case the noun usage is more common). The exposure sentences biased towards the dispreferred usage and at test participants were confronted with the isolated ambiguous words and asked to form a sentence using that word, so that its word class in the sentence could be determined. Just as with word-meaning, word-class priming was observed after a delay of about 20 min, and this effect of exposure to the biasing sentences remained stronger after sleep than after wake. The balance of evidence then is consistent with word-meaning (both specific and generalised), word-class, and word-sense priming remaining strong after short periods awake, or longer periods asleep, but diminishing across time spent awake prior to sleep. This fits well with the idea that language comprehension is routinely accompanied by the encoding of sentential material in episodic memory, although this encoding is likely to be abstracted away from the surface detail of the words in the sentence.

It is likely that what is being encoded in the experiments just described is strongly related to the process of extraction and retention of event knowledge from linguistic material (Cohn-Sheehy et al., 2022; Zacks, 2020; Zwaan & Radvansky, 1998). This link is further supported by evidence that the hippocampus may be involved in identification of event boundaries during perception of naturalistic events (Ben-Yakov & Henson, 2018) and language (Baldassano et al., 2017; Bilkey & Jensen, 2021). That said, although the hippocampus is seen as central to tracking of event boundaries, there is no consensus on the extent to which consolidation is involved in the retention of linguistic events such as sentences (Cohn-Sheehy et al., 2022; Denis et al., 2021). In attempt to address this question, Mak et al. (2023b) ran a comprehensive study of retention of story material across 12 hr of sleep or wake. The results were mixed, in an interesting way. In a recognition paradigm intended to assess different levels of abstraction for sentence memory (Fisher & Radvansky, 2018), no differences between sleep and wake were found at any level of abstraction. Perhaps surprisingly, no sleep effects were seen in a free recall task either. The only task that revealed sleep benefits over wake (in two experiments) was one in which participants were required to fill in key missing words in a sentence frame. For this task, veridical memory for the words was weak and not affected by the sleep/wake manipulation, but errors tended to be more closely related to the intended word for the sleep condition compared with the wake condition. The conclusion from this study is that consolidation benefits in discourse memory might be quite specific. The “fill in the blank” task was in some ways similar to the word-association test in word-meaning priming in that the specific association between a target word and its context was being tested. In these circumstances, the associative links between the various words in a sentence need to be retained, and potentially episodic memory and consolidation are brought in to play. In other circumstances, potentially, cortical schematic knowledge is brought to bear on the retention of text, meaning that consolidation effect will be absent or at least less obvious.

## An integrated framework for word learning, recognition, and interpretation

**Figure 2.**
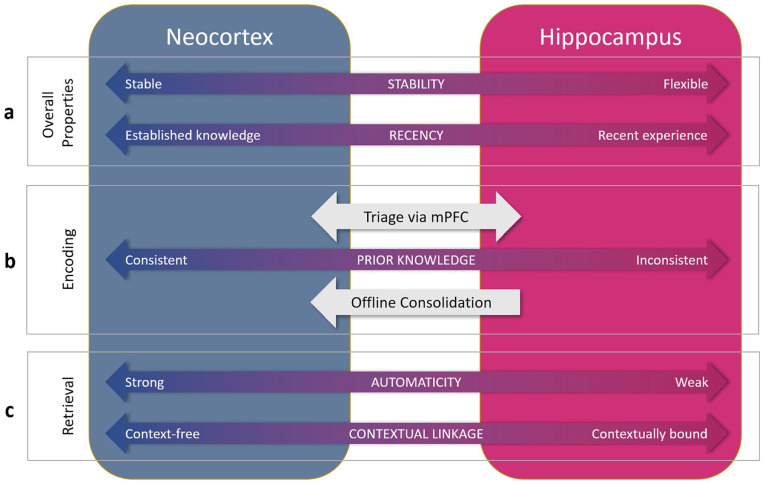
Organisational dimensions relating to word learning, recognition, and interpretation along the two pathways of a complementary systems account. a. Neocortical representations tend to be stable and well-established representations, whereas hippocampal representations will be flexible and reflect recent learning. b. Encoding will depend on consistency or overlap with prior cortical knowledge (tested via mPFC triage), with consistent material more likely to be cortically encoded. Prior knowledge-inconsistent material (e.g., arbitrary mappings) will depend more on hippocampal mediation at encoding, and on subsequent consolidation to the cortical pathway. The hippocampal encoding and subsequent consolidation route is likely to be the main means of encoding, both of novel words and of existing words when encountered in sentence context. That said, encoding using both routes to differing extent is possible. c. Retrieval of neocortical lexical knowledge will be strongly automatic (e.g., fast, obligatory), whereas hippocampal retrieval will show weaker properties of automaticity. Likewise, neocortical knowledge will be less context-dependent than hippocampal lexical knowledge. In many cases, retrieval will involve the combined knowledge stored in both pathways, particularly when there is recent relevant experience of one or more words in the sentence.

My thinking in relation to the neurocognitive architecture underpinning psycholinguistic processing of words can be seen as a gradual shift between three stages, varying in the breadth of properties accommodated. The focus has been on spoken language, but much of the resulting theory can be reasonably interpreted as applying to written language as well at least in broad terms. In even broader terms, some of the principles likely also apply to production (cf. Dell et al., 2021; Gaskell et al., 2014), but we will maintain focus on comprehension at this point. With William Marslen-Wilson, we developed a distributed connectionist model of word recognition (Gaskell & Marslen-Wilson, 1997) that accommodated many properties of earlier models (e.g., Marslen-Wilson & Tyler, 1980; McClelland & Elman, 1986), but instantiated in a distributed neural network framework. However, when applied to the question of how to learn and lexically incorporate new words, this framework turned out to be insufficient, and so with Matt Davis, we proposed a complementary systems account of word learning (Davis and Gaskell, 2009), embedding the original model as the cortical component of a dual-pathway network, with a second, hippocampal pathway to allow swift incorporation of new words. This broader model helped to explain a now substantial and reasonably consistent body of data (cf. Schimke et al., 2021) that suggested that word learning involves a process of sleep-associated consolidation to fully establish new words in the mental lexicon. Subsequent empirical evidence has also shown that a stark division between a stable cortical route dedicated to word recognition and a flexible hippocampal route dedicated to word learning is untenable. The full picture that emerges from the evidence described in this article is that both pathways contribute to both learning and recognition, but not equally, and depending on certain properties. In Figure 2, I lay out some of the key properties that characterise the two pathways, in terms of the way in which they both learn in response to novel words and retrieve stored lexical knowledge during word recognition. My view is that the lexicon can only be fully understood through interrogation of both pathways and to my knowledge this represents a divergence from all current models of word recognition, which (using the terminology of this article) focus on the stable route in explaining how we recognise words.

This would be a justifiable simplification if the only involvement of the flexible pathway was for learning and binding of cortical information for new words. Although adults encounter novel words almost on a daily basis, nonetheless, the vast majority of words that are encountered during adulthood are well-established and familiar ones, and so would have little need of the flexible pathway. However, the third stage of my development of understanding is that the flexible pathway does more than just represent new linguistic material such as words. Building on Jenni Rodd’s word-meaning priming research (e.g., Gaskell et al., 2019), we have made the case that in fact the flexible pathway helps to support the tracking of contextual information about recently encountered words, providing a secondary source of knowledge to aid interpretation of those same or similar words at a later point. This shift in thinking does not involve any changes to the fundamental architecture of the complementary systems account (see Figure 1), but does imply a much more substantial role for the flexible pathway in language comprehension. In its strongest form, the episodic context account proposes that every new sentence or utterance is an opportunity to encode a new representation in episodic memory, and this new memory may contribute to later understanding, or be consolidated into the stable pathway, or be forgotten, depending on a range of factors. Of course, this characterisation may be too strong, and indeed there may well be limits to the circumstances in which comprehending a sentence entails encoding it, but here we are restricted by the body of evidence so far. Nonetheless, on the basis of recent studies (Curtis et al., 2022; Mak et al., 2023a, 2023b), our best guess is that episodic encoding and consolidation is a fairly common part of everyday comprehension. This characterisation of the act of comprehension as an opportunity for learning is not in itself a novel idea. Indeed, many connectionist approaches to language learning are built on this tradition (e.g., Elman, 1990; Gaskell & Marslen-Wilson, 1997; Seidenberg & McClelland, 1989). However, such accounts have relied on small and gradual weight changes to support learning. The difference here is the extension to a complementary systems account, which provides an opportunity for more substantial one-shot learning effects, along with the argument that these encoding opportunities are a routine part of comprehension rather than the exception. Interestingly, one of the key advances that has led to the remarkable recent successes of large language models in artificial intelligence (AI) approaches to engineering is exactly the method advocated here. As discussed by Botvinick et al. (2019), recent reinforcement learning approaches to AI have mitigated the substantial training costs of incremental deep learning by exploiting a form of episodic memory that allows fast learning to interact with the incremental network. In effect, AI exploited to the full a computational trick borrowed from cognitive science. The approach I advocate for lexical processing allows AI to return the favour (cf. Lu et al., 2022).

If normal language comprehension is characterised as a combination of a decoding process and an episodic encoding opportunity, a crucial question then is what benefits does the encoding (and possibly subsequent consolidation) provide? At least three potential benefits can be considered. The first is Rodd’s original explanation for the observation of word-meaning priming (Gilbert et al., 2018; Rodd et al., 2013): that encoding and consolidating recent interpretations of words helps to ensure that their lexical representations reflect the statistics of language. In the case of homonyms, this most obviously applies to the balance between their different meanings, but the argument can be made more generally that all words have rich semantic representations for which any particular instance will need only a selection of those properties (e.g., Johnson-Laird, 1987).

A second argument is that more recent interpretations of words will be more relevant to future comprehension than older interpretations. This is because events in a wide range of human actions do not have a truly random distribution (e.g., library visits, stock trades, email exchanges, Barabási, 2005; Vázquez et al., 2006). Instead, there are bursts or flurries of similar actions with brief intervals, followed by longer periods with very few or no related actions. Myslín and Levy (2016) showed that a similar burstiness is seen in a linguistic corpus analysis for sentential complements, and it would be remarkable if the same non-random properties were not seen for lexical items generally and more specifically their interpretations. As a concrete example, if you are reading a book and encounter the word *pen* in its “animal enclosure” interpretation early on, there is a better than base rate chance that the next instance of that word will have the same interpretation. Expectation of repetition is a rational response to this observation about the world (Myslín & Levy, 2016), and the contextual encoding of words during sentence comprehension provides a mechanism by which this expectation can be implemented. Interestingly, this particular benefit might explain why word-meaning priming effects are typically much stronger than might be expected if the objective is simply to update the overall lifetime statistics of an ambiguous word. Speculatively, a strong initial episodic contextual representation of an instance of a particular word can support the time-limited expectation of repetition (via the flexible pathway), but trace decay during wake and subsequent consolidation of whatever is left of the episodic trace prior to sleep can lead to a weaker (and more representative) effect of the new instance on the long-term expectations about that word via the stable pathway. Furthermore, the ability to capture the burstiness of language may be important for long-term retention of vocabulary. Cychosz et al. (2024) measured the burstiness of child-direct speech for children aged 2–7. They found that children who experienced more bursty language tended to have larger vocabularies, and this burstiness was a stronger predictor of vocabulary size than overall input quality.

A final potential benefit is a means of retaining linguistic experiences to inform later actions and conversations. We need to preserve at least some of our knowledge of conversations and texts over minutes, hours, days, months, and (potentially) years. Admittedly, the evidence that sleep supports the maintenance of memory for linguistic material is slight, and perhaps suggests that episodic encoding is one of several memory resources that can jointly support long-term retention, in the case of episodic memory, particularly reflecting the contextual associations in the text. A key uncertainty here relates to the extent of any episodic contribution to the formation and maintenance of event representations or situation models during language comprehension (Zwaan & Radvansky, 1998).

Returning briefly to the literature on hippocampal amnesia, one implication of the account I have described is that damage to the hippocampus would lead to weaknesses in keeping track of word meanings in the short term to support expectation of repetition, and in the longer term to maintain memory of conversation and update/optimise meaning frequency ratios. Although to date word-meaning priming has not been tested in participants with amnesia, there is less direct evidence that pertains to these issues. Certainly, it is well-established that verbal material is relatively poorly retained in amnesia. For example, when tested after an hour on recall of sentence-final words (e.g., “At the fair, Sarah lost her *keys*”) using the preceding context as a cue, amnesia patients had substantially lower retention levels than their control group, with performance more similar to control performance at a delay of 1–2 weeks (Shimamura & Squire, 1988). Recognition memory was also disadvantaged, although performance was well above chance level. This evidence fits with an important, but not exclusive role of the hippocampus in retention of verbal material.

At a shorter timescale, language processing often relies on linking material across separate sentences to integrate understanding and resolve ambiguity. For example, successful interpretation of the pronoun *she* in “She is wearing a blue dress” may require memory of a previous sentence referring to a female character. Kurczek et al. (2013) tested participants’ ability to resolve these ambiguities, comparing younger and older healthy controls with hippocampal patients and a brain-damaged comparison group. Unlike all the other groups, the hippocampal patients showed substantial disruption to the pronoun resolution process, at least in cases where the resolution was made more difficult by the prior introduction of two female characters, introducing an ambiguity to resolve. Thus, although much of sentence comprehension is unaffected by damage to hippocampal structures (e.g., Brown-Schmidt et al., 2021), maintenance of key information across sentences seems to be somewhat impaired. This is exactly what would be needed to allow an expectation of repetition as described above.

Finally, in terms of the ability to maintain and update semantic representations of words, again hippocampal patients appear to have deficits. This deficit is observed in tests that address the richness of semantic representations of words, such as the generation of associates, senses, or semantic features (Klooster & Duff, 2015), with those features tending to be closer to the target words in semantic space (Cutler et al., 2019). Consistent with the arguments made in this article, Klooster and Duff interpreted their finding in terms of a protracted role for maintaining and enriching semantic knowledge through the integration of new information via discourse.

The integrated dual-pathway framework in Figure 2 has important aspects that remain uncertain. This is particularly evident for the most recent development of the framework proposing an involvement of the flexible episodic pathway in encoding and comprehending familiar words in sentence or utterance context. One key question is “how episodic is episodic”? In other words, how contextually detailed are the representations that are encoded during language comprehension? In the domain of spoken word recognition, there is a substantial literature that points to quite detailed information about the talker that can be retained in memory during word perception. This information can lead to talker-specificity effects at a later point when the same word is processed, depending on whether the talker at test matches the talker at encoding (see Tenpenny, 1995, for a review). These effects tend to fade over time (Goldinger, 1996), although if the level of exposure is repeated and sufficient, they can still be observed at least a week later (Brown & Gaskell, 2014; Ernestus, 2009). In contrast, word-meaning priming shows no evidence of talker-specificity (Rodd et al., 2013, 2016) and transfers well between spoken and written modalities (Gilbert et al., 2018). Indeed, this was one of the reasons why word-meaning priming was seen as not relying on episodic memory. Furthermore, word-meaning priming can be observed when the target word is replaced at exposure by a near-synonym (Curtis et al., 2022).

All of these pieces of evidence point to word-meaning priming occurring at a level of abstraction not seen in the literature on repetition priming for words. However, it is possible that episodic memory is flexible enough to prioritise different levels of representation depending on the nature of the experience and the exigencies of the situation. When confronted with a list of isolated spoken words that may vary in the talker, the finer details of the speech signal such as talker identity may be salient to the participant and so attended to during encoding. On the contrary, when listening to meaningful sentences with an immediate requirement to comprehend and judge meaning relationships, these fine details may be deprioritised, with the semantic relationships between the words featuring more prominently in episodic memory. This would be a view of episodic memory more in line with the episodic memories that are needed in studies of sleep and memory consolidation (e.g., paired-associate learning). In such tasks, there is often an explicit requirement to form a vivid mental image or story that can link the two elements in each pair (Cairney et al., 2018). There are further parallels here with the event models that comprehenders naturally form when listening to linguistic material (e.g., Richmond & Zacks, 2017).

It is also important to note that the evidence base that supports the involvement of the flexible pathway in comprehension is meagre compared with that relating to word learning. In particular, neuroimaging evidence implicating the hippocampus in word-meaning priming is absent (although see Blank & Fedorenko, 2020; Chen et al., 2016 for evidence relating to interactions between the hippocampus and the default mode network in longer-term retention of linguistic material), and this would be an important test of the predictions of the account I have described. The evidence relating to word learning also suggests that the flexible and stable pathways are jointly involved in learning and processing of new words, and it may turn out that retention of sentential information is similarly supported by the two pathways jointly, or that their relative involvement depends on the nature of the material.

## Conclusion

In this article, I have provided an update on the role of complementary systems in the learning and processing of words during comprehension. There is now a solid body of evidence consistent with a flexible pathway to lexical knowledge that most likely relies on episodic memory and the hippocampus to some extent, and provides an opportunity for later consolidation in sleep and other offline states. This complements a more stable, cortical route to lexical knowledge that was perhaps traditionally thought of as the lexicon “proper.” Sleep-associated consolidation effects are well-established, but in this article, I have tried to emphasise that the complementary nature of the two pathways does not imply a dichotomy, with learning exclusively involving the flexible route and comprehension involving the stable route. Rather, we see that both routes are involved in both aspects of word processing, with various factors determining the level of involvement of the stable route for learning and the flexible route for processing. Furthermore, I have argued that the remit of the flexible pathway in learning goes beyond an initial means of dealing with novel lexical items. Although further evidence is needed here, it may be that the flexible pathway is routinely involved in comprehension of language in that it allows contextual learning of words, such that listeners can deal optimally with the non-random distribution of language, update long-term lexical knowledge, and help to maintain lasting memory of linguistic events.
